# Smartphone-Based Dual-Modality Imaging System for Quantitative Detection of Color or Fluorescent Lateral Flow Immunochromatographic Strips

**DOI:** 10.1186/s11671-017-2078-9

**Published:** 2017-04-21

**Authors:** Yafei Hou, Kan Wang, Kun Xiao, Weijian Qin, Wenting Lu, Wei Tao, Daxiang Cui

**Affiliations:** 1 Department of Instrument Science and Engineering, School of Electronic Information and Electrical Engineering, Shanghai Jiao Tong University, Shanghai, 200240 China; 2 Shanghai Engineering Research Center for Intelligent Diagnosis and Treatment Instrument, Shanghai, 200240 China; 3 Zhujiang Hospital, Southern Medical University, 253 Gongye Road, Guangzhou, Guangdong 510280 China

**Keywords:** Smartphone, Dual-modality imaging, Lateral flow strip, Quantitative detection

## Abstract

Nowadays, lateral flow immunochromatographic assays are increasingly popular as a diagnostic tool for point-of-care (POC) test based on their simplicity, specificity, and sensitivity. Hence, quantitative detection and pluralistic popular application are urgently needed in medical examination. In this study, a smartphone-based dual-modality imaging system was developed for quantitative detection of color or fluorescent lateral flow test strips, which can be operated anywhere at any time. In this system, the white and ultra-violet (UV) light of optical device was designed, which was tunable with different strips, and the Sobel operator algorithm was used in the software, which could enhance the identification ability to recognize the test area from the background boundary information. Moreover, this technology based on extraction of the components from RGB format (red, green, and blue) of color strips or only red format of the fluorescent strips can obviously improve the high-signal intensity and sensitivity. Fifty samples were used to evaluate the accuracy of this system, and the ideal detection limit was calculated separately from detection of human chorionic gonadotropin (HCG) and carcinoembryonic antigen (CEA). The results indicated that smartphone-controlled dual-modality imaging system could provide various POC diagnoses, which becomes a potential technology for developing the next-generation of portable system in the near future.

## Background

In recent decades, lateral flow immunochromatographic strip (LFICS) has been increasingly applied as a diagnostic tool for point-of-care test (POCT) because of its simplicity, rapid speed, sensitivity, and specificity [1]. Nowadays, this technology has been applied for many areas such as food security, environment monitoring, and drug testing [2–8]. In order to meet the requirement of different detection, diverse signal marker strips had been developed, including colloidal gold strip, magnetic nanoparticles strip, and fluorescence strip [9–12]. As the typical nanomaterials, gold nanoparticles (Au NPs) have the color-tunable property with size, which also can be functionalized with peptides to avoid degradation in order to facilitate to finish quick immunological reaction and display different degree of color for half-quantitative detection [1, 11, 13]. Because of their unique advantages, such as simplicity, rapidity, and ease of interpretation, Au NPs-based LFICS was widely used for rapid diagnosis [1, 11, 13]. Magnetic nanoparticles (MNPs) could be easily gathered through directing external magnetic field exposure; thus, the targeted molecule modified on the MNPs could be detected even with less amount used [14]. Magnetic signals could be entirely captured by the devices so that MNPs-based LFICS realized quantitative measurement, thereby increasing LFICS sensitivity [12, 14, 15]. Fluorescent NPs possessed many advantages, including photo-stability, wide absorption and narrow emission spectra, more noticeable size-dependent Stokes shifts, robust stability against photo-bleaching and chemical degradation, higher fluorescent quantum yield, high sensitivity, and large molar extinction coefficients [16–18]. These excellent properties rendered fluorescent NPs playing an important role for developing highly sensitive LFICS. Different kinds of fluorescence LFICS were studied, such as testing chloramphenicol, nitrated ceruloplasmin, ochratoxin, alpha fetoprotein [16–20].

Until now, the typical detection based on LFICS is through the naked eye, which was widely used in food safety, environment monitoring, and precision medicine. However, this detection can only provide qualitative test (positive or negative) or semi-quantitative information on analyte concentration, which the LFICS could not satisfy requirements for practical applications. Therefore, many devices providing quantitative analyte concentration for testing LFICS had been developed [9, 21]. Some strip readers were designed to work in the desktop computer or laptop, which possessed rapid processing speeds and stable performances. However, these bulky and heavy devices limited their wide application for the trend of family and personalize care. Mei’s group developed an embedded system based on the Acorn RISC Machine (ARM) processor, which applied for reading a test strip [22]. Marquina’s group designed a spin-valve giant magnetoresistive (GMR) sensor system to quantify the amount of analyte, which were not portable for outdoor detection [23]. Obviously, the strip reader based on mobile device could be satisfied with the requirement of high portability and feature-rich testing. As a matter fact, the mobile health market is rapidly developing and portable diagnostics tools provide an opportunity to increase the availability of healthcare and decrease costs [24]. Therefore, various strip readers based on mobile device have been developed. For example, Oncescu’s group developed a smartphone accessory and software application based on smartphone that allowed for the quantification of cholesterol levels in the blood [25] and Hyun Park’s group developed a kind of strip reader for quantitative measurements of H5N1 using fluorescent strips [26]. Meanwhile, some other strip readers based on smartphone were obtained, such as testing thyroid-stimulating hormone, cholesterol, malaria, and tuberculosis [9, 27–32]. However, all the strip readers based on smartphone only can detect one type of strips from the color or fluorescence which limited their multifunctional applications [33–36].

In this study, smartphone-based dual-modality imaging system was developed, which could quantitatively detect color or fluorescent lateral flow immunochromatographic strip (ICTS). In this system, the white and UV light of an optical system was designed and could be changed according to the different kinds of strips (color strip or fluorescence strip). The improved Sobel operator was used in the software, which improved greatly the ability of distinguishing between the test area and background boundary information. This smartphone provided the power to the whole system and could be continuously operated for 4–5 h.

The detection limit of the reader reached the requirements as the limit values were 2.3 mIU/mL and 0.037 ng/mL separately, which were calculated separately from detection of color strip (human chorionic gonadotropin, HCG) and fluorescence strip (carcinoembryonic antigen, CEA). To our knowledge, it is one kind of novel dual-modality imaging for immunochromatographic strip based on smartphone and has great potential in food security, environment monitoring, and drug testing in near future.

## Methods

### Composition of Test Strips

**Fig. 1:**
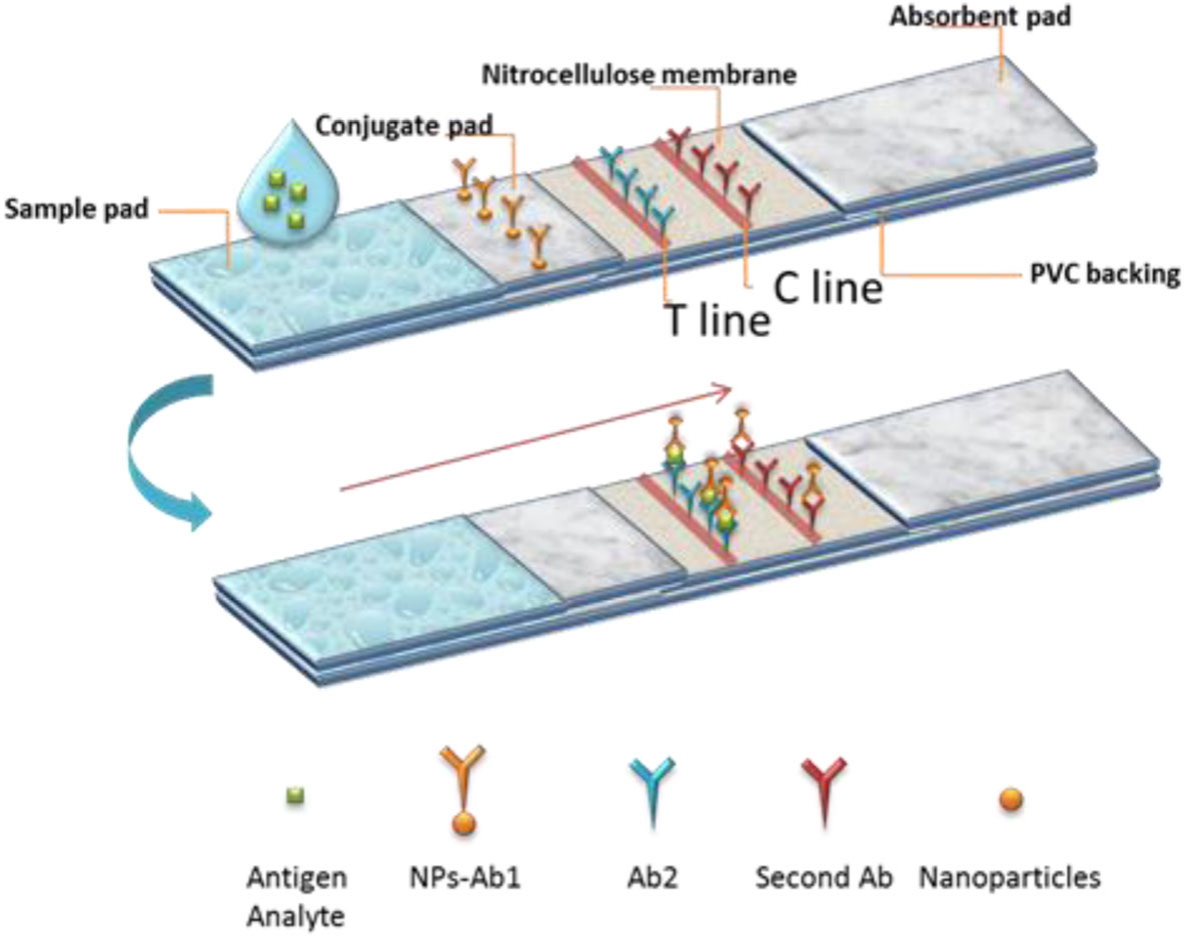
The schematic image of the configuration and the principle of the detection

The antigen and antibody of the HCG and CEA were both purchased from the Shanghai Linc-Bio Science Co. LTD (shanghai, China). Conjugate, sample, and absorbent pads as well as the nitrocellulose (NC) membrane and polyvinyl chloride (PVC) plate were purchased from JieYi Biotech Co., Ltd. (Shanghai, China). Immunochromatographic strips were prepared by our group’s member. The test strip was composed of a sample pad, conjugate pad, and absorbent pad, as well as a nitrocellulose (NC) membrane and PVC backing card, as shown in Fig. 1. All pretreated parts were assembled sequentially onto a PVC backing card with 2 mm overlap of each component. The assembly was cut into 3-mm-wide individual strips and then stored at 4 °C inside a sealed plastic bucket with a desiccant until further use.

Primary antibody (Ab2) against the antigen was immobilized on the test line (T line), and another antibody (Ab1) against a different epitope of the same antigen was labeled with NPs. When the NP-labeled antibody flowed along with the test sample (which was expected to have the antigen), it interacted with the antigen in the sample. This complex interacted with the antibody coated at the test line on the membrane and formed a sandwich (Fig. 1). A control line was fixed with the second antibody against the primary antibody, which captured the remaining antibody labeled with NPs. Thus, in a sandwich assay format, a positive test sample is indicated by the appearance of two colored lines (T line and C line) and a negative test sample was indicated by the appearance of one colored line that is only the control line (C line).

### Design of Hardware

**Fig. 2:**
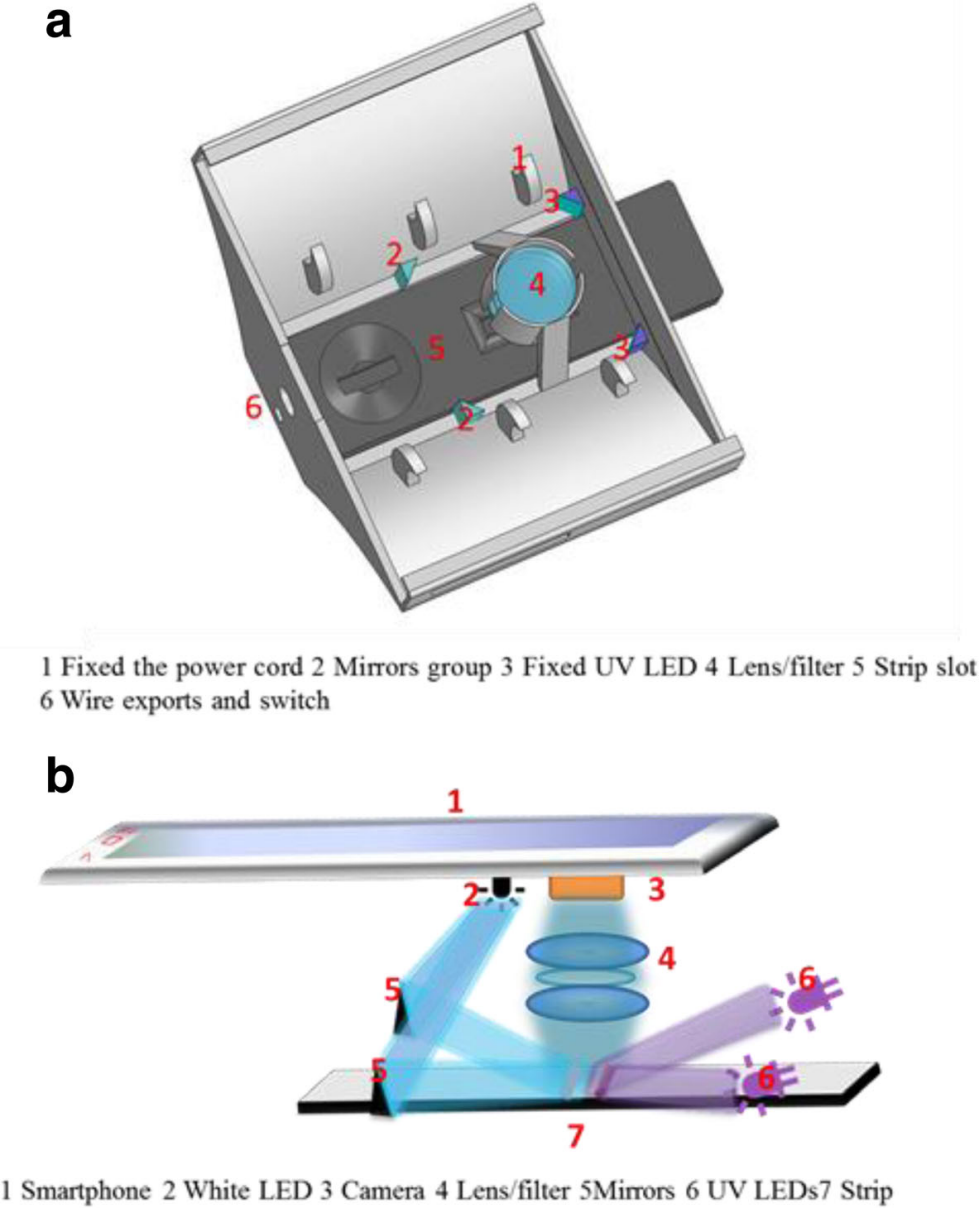
**a** The closed shell module. **b** The work schematic of the smartphone detector device and schematic illustration of dual-modality imaging system. When color strips were test, the white LED turned on and the UV LED turned off. While the UV LED turned on, the fluorescent strips were to be tested

Dual-modality imaging system was developed based on smartphone. In order to decrease the cost and increase the cost performance, Red Mi Note 2 smart phone (Xiaomi mobile internet company) was chosen as the main part. The smartphone was equipped with Central Processing Unit (CPU, MediaTek Helio X10 MT6795 processor) and Graphics Processing Unit (GPU, PowerVR G6200), and its processing speed and computing performance were greatly for image handling. Even though this smartphone screen has a high resolution (1920 × 1080 p) and the screen pixel density is 441 ppi. The rear camera is based on complementary metal-oxide-semiconductor (CMOS) image sensor with 13 million pixels. Therefore, using smartphones as a data acquisition, image processing, and light module was appropriate. Black smartphone accessory (55 × 78 × 30 mm^3^) and an ICTS cartridge (70 × 18 × 4.8 mm^3^) were designed by SolidWorks software and fabricated using a 3D printer (Fig. 2a). The attachment module included optical path structure, lens, filter, sample slot, LED, and some fixed structure. Two ultra-violet (UV) LEDs (365 nm, 1 W) were fixed on both sides of the upper end of the cartridge, and the flash of smartphone was selected as the white light. The filter and lens group has been fixed in the accessory box. Appropriate design of optical paths (Fig. 2b) not only provided homogeneous optic field but also shortened the focusing distance, which helped us get the high-quality images within just 20-mm height shell. Dual-modality imaging (using white LED or UV LED) can be switched easily according to different kinds of strip (colloidal gold strip and fluorescence strips). The smartphone provided power to the whole system and could be operated continuously in 4–5 h.

### Software Features

A complete dual-modality imaging system required not only hardware but also powerful and easily operated software. The Java programming language was employed in the design of Android Studio 1.5 development environment and provided main functions of processing test strip images, analysis, and diagnosis in the smartphone. The system has the features of accuracy, friendly and mostly. The dual-modality imaging system not only can test the colloidal gold strips through the colorimetry but also can test the quantum dot strips through the florescence signal.

**Fig. 3:**
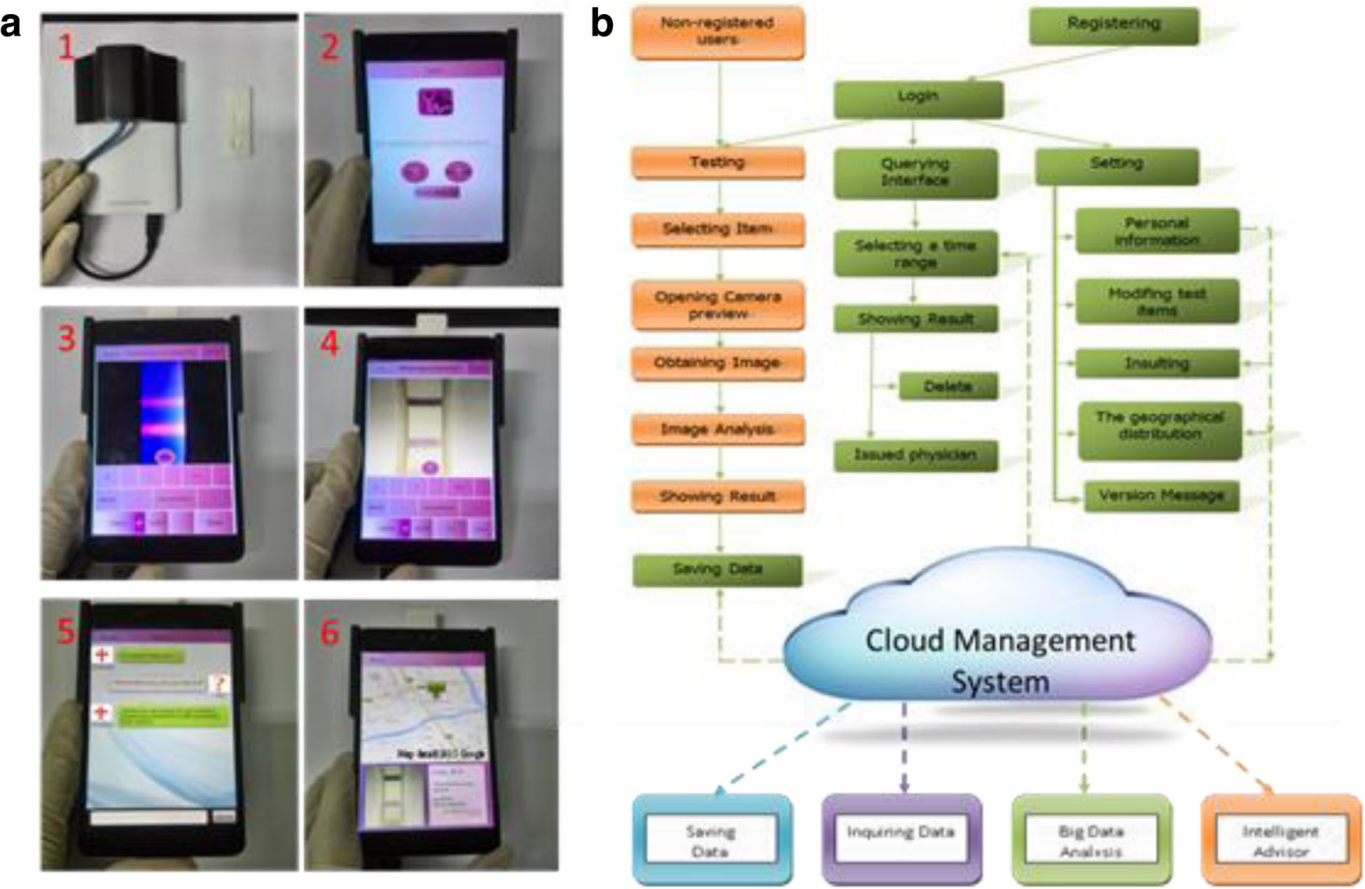
**a** *1* Back view of testing equipment and the strip shell physical figure. *2* The main menu of the software. *3* The fluorescent strip test displaying (CEA). *4* The color strip test displaying (colloidal strip for testing HCG). *5* The robot service through chat. *6* The location distribution of the test result in Google map (Map data: Google, DigitalGlobe). **b** Flow diagram of the device software

The function of software included the detection and analysis of cloud data, cloud data storage, cloud data queries, intelligent robot service consultant, and location of the user. After the strip is inserted, the user could choose the detection mode (UV or white LED) to open the camera and click the test button, and then get the analysis results while waiting for 3 s. All the result data would be stored in the cloud and could be queried when the user needed them. Through the system, the user could get the geographical distribution of the patients by analysis the cloud data. In the system, the intelligent robot consultant was also developed, which could provide some useful messages according to the user’s questions, see in Fig. 3a. In the flow diagram of the software system in Fig. 3b, the orange color part was used for the non-registered patients, and the test results still could be saved in our cloud data manager system. The green flow chart is used for the registered users. Once registered, the users can get much more permission to access the software function, such as managing their own detection results and getting the advices from the intelligent robot consultant.

In Fig. 3b, it showed that saving and inquiring data were the based function which was for upholding the software, and then, the big data analysis and the intelligent advisor was the advance function. Through the data analysis, the user distribution was easily viewed on the map, which can provide government disease control. And through the intelligent advisor, the users could get some useful advices according their own healthy condition. With the rapid development of big data and cloud storage, clinical monitoring data contains a great value. In order to take advantage of excavation clinical data value, we developed a corresponding cloud data management system which emerged in the dual-modality imaging system. The system could achieve high-volume statistical data analysis, query, and management and could be classified according to the statistics geographical distribution of the disease, which would provide a reference for the epidemiological investigation.

### Clinical Specimen Examination

This study was approved by the Medical Ethics Committee of Shanghai Jiao Tong University, China. All patients signed an informed consent, and all methods were performed in accordance with the relevant guidelines and regulations. All the clinical samples were collected from Shanghai Ninth People’s Hospital, the affiliated hospital of Shanghai Jiao Tong University. Total 50 urea-enzymes samples and 50 antigens of CEA were tested using a dual-modality imaging system employing lateral flow strips.

### Data Statistic

All data are presented in this paper as mean ± standard deviation. Statistical differences were evaluated using the *t* test and considered significant at *P < 0.05*.

## Results and Discussion

**Fig. 4:**
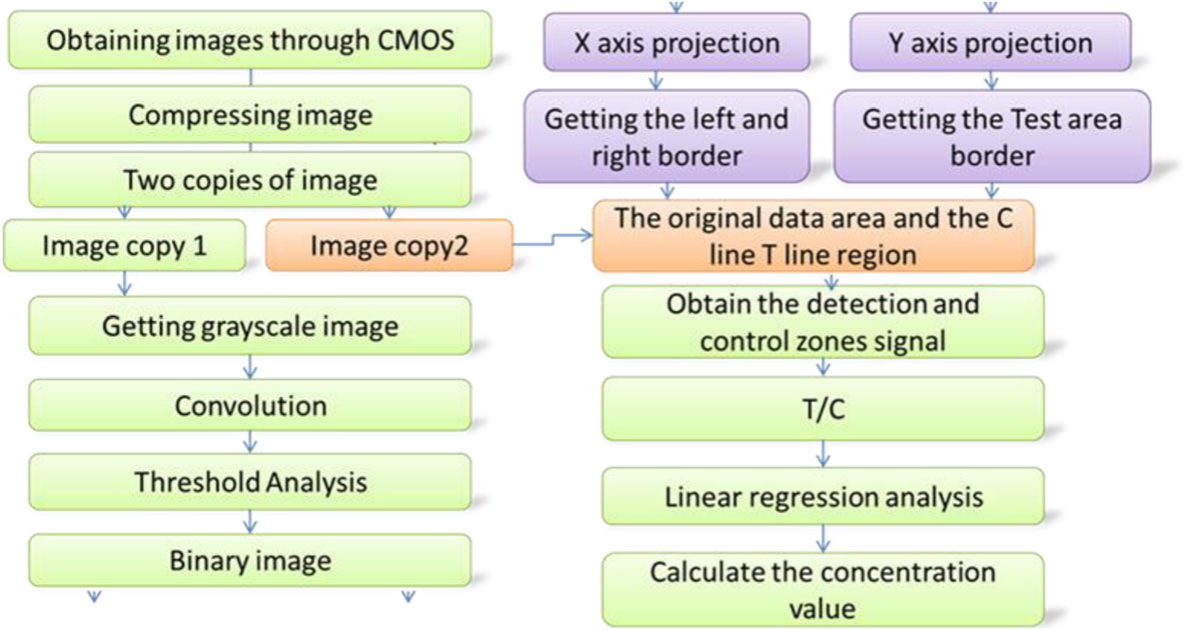
The flow of the algorithm

To produce accurate diagnosis, a high-resolution image was acquired. The camera in the smartphone was based on CMOS image sensor with 13 million pixels. It has excellent optical character, high purity, whiteness, high resolution, and superb color rendition. In the dual-modality imaging system, the camera could be operated conveniently by clicking the control button. In order to obtain more accurate signal data, multiple image processing was applied, such as gray-scale processing, improved Sobel convolution operator, threshold analysis, image binarization, and getting detection area boundary coordinates, as shown in Fig. 4. All the images were captured every 0.1 s and three times in total to obtain the average value.

### Research of image edge examination

Once the image data was obtained, the signal area in the image was then identified. Compared with many other algorithms [6, 28–31], this algorithm could be easily be used in smartphone. Converting the three-channel image into a single-channel grayscale image mainly simplify the calculation of subsequent processing on gray-scale process. In this case, it is very convenient to find out the feature boundary of the detecting area. Sobel convolution operator algorithm was proposed to process the image and advantaged to search the boundary of T and C lines. The procedure of the proposed algorithm is listed below.

1. *Sx* is the *x* direction calculation result. *Sy* is the *y* direction calculation result. *S(x*, *y)* is the result of convolution operation after the *f(x*, *y)* function.
  1 $$ S x=\left[3 f\left( x+1,\  y\mathit{\hbox{-}}1\right)+10 f\left( x+1, y\right)+3 f\left( x+1, y+1\right)\right]\mathit{\hbox{-}}\left[3 f\left( x\mathit{\hbox{-}}1,\  y\mathit{\hbox{-}}1\right)+10 f\left( x\mathit{\hbox{-}}1,\  y\right)+3 f\left( x\mathit{\hbox{-}}1,\  y+1\right)\right] $$
  2 $$ S y=\left[3 f\left( x\mathit{\hbox{-}}1,\  y\mathit{\hbox{-}}1\right)+10 f\left( x, y\mathit{\hbox{-}}1\right)+3 f\left( x+1, y\mathit{\hbox{-}}1\right)\right]\mathit{\hbox{-}}\left[3 f\left( x\mathit{\hbox{-}}1,\  y+1\right)+10 f\left( x,\  y+1\right)+3 f\left( x+1,\  y+1\right)\right] $$
  3 $$ S\left( x, y\right)=\sqrt{\left( Sx\hat{\mkern6mu} 2+ Sy\hat{\mkern6mu} 2\right)} $$
2. Matrix is shown below in (4), which was Sobel mask operator. The matrix was used in Eqs. (1) and (2).

4 $$ \left[\begin{array}{c}\hfill -3\hfill \\ {}\hfill -10\hfill \\ {}\hfill -3\hfill \end{array}\kern0.24em \begin{array}{c}\hfill 0\hfill \\ {}\hfill 0\hfill \\ {}\hfill 0\hfill \end{array}\kern0.24em \begin{array}{c}\hfill 3\hfill \\ {}\hfill 10\hfill \\ {}\hfill 3\hfill \end{array}\right],\left[\begin{array}{c}\hfill -3\hfill \\ {}\hfill 0\hfill \\ {}\hfill 3\hfill \end{array}\kern0.24em \begin{array}{c}\hfill -10\hfill \\ {}\hfill 0\hfill \\ {}\hfill 10\hfill \end{array}\kern0.24em \begin{array}{c}\hfill -3\hfill \\ {}\hfill 0\hfill \\ {}\hfill 3\hfill \end{array}\right] $$

For the boundary line of the acquisition, the use of Sobel operator was much better in comparison with that of other edge detection operators.

**Fig. 5:**
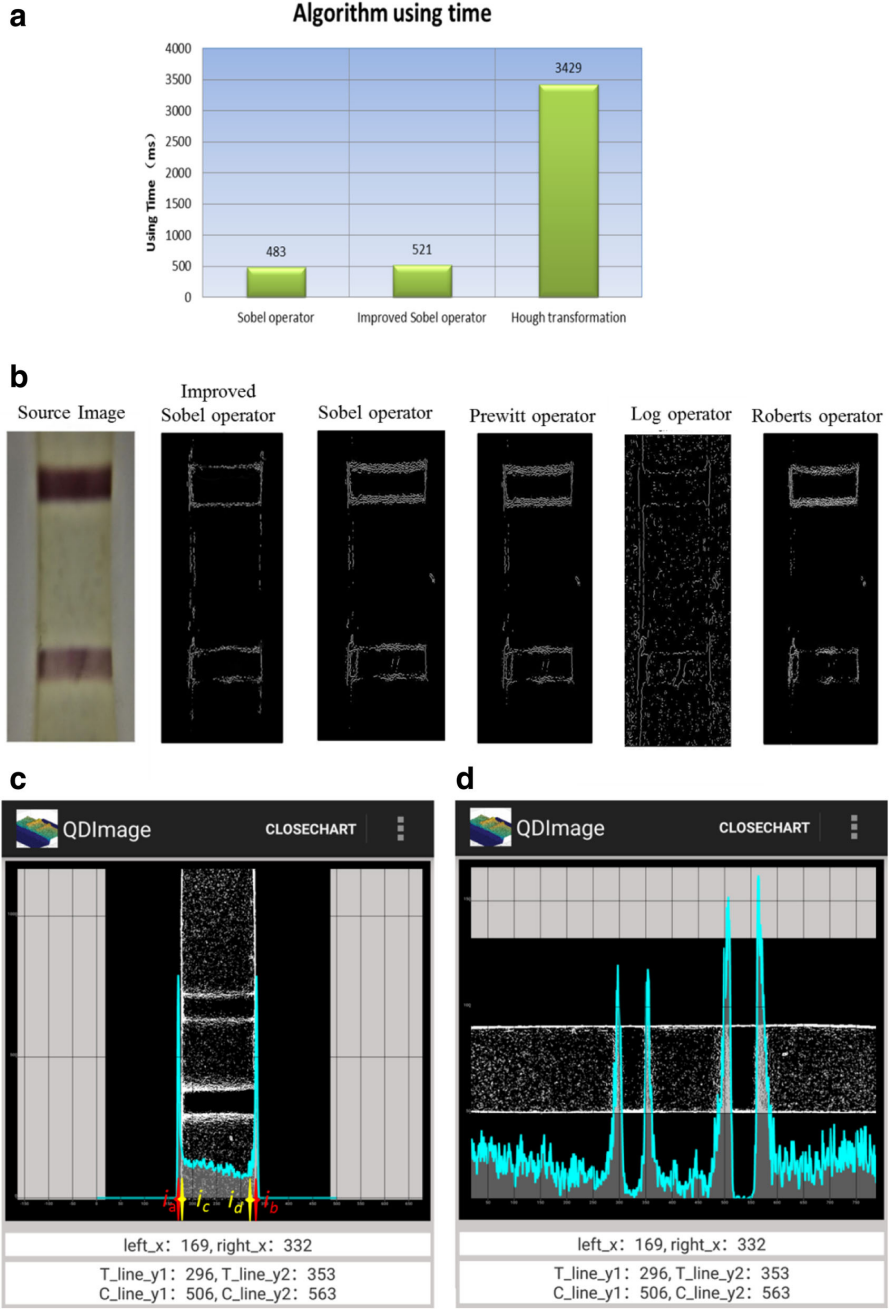
**a** Comparison of three time-consuming algorithms (Sobel algorithm, improved Sobel convolution operator, and Hough transformation). **b** Binarization images through five algorithms (improved Sobel convolution operator algorithm, Sobel algorithm, Prewitt algorithm, Log algorithm, and Roberts algorithm). **c**, **d** The smartphone screenshots which displayed the boundary coordinates. **c** Two methods for obtaining the *left* and *right* coordinates (*left*_x and *right*_x the same as the *ic* and *id*). **d** The coordinates of the test line and control line (T_line_y1, T_line_y2, C_line_y1 and C_line_y2 same as the t1, t2, c1 and c2). The paint software was developed by our research group in android operating system

Besides, even though there are many advantages for linear detection with use the typical way of Hough line detection algorithm, the strip mainly composes of straight lines but software needs much more time for using this algorithm, which limited its use in this technology. Figure 5a showed the time-consuming algorithm comparison.

The next step was to select the gray image threshold before it was binary processing. It could acquire a much better accurate threshold value after combining the optimal threshold segmentation principles and experience value. The effect of the optimal threshold method alone was acceptable, yet it was also a more time-consuming algorithm. To avoid that drawback, it was a fast and simple way to combine the algorithm and the reference value being stored in the database to calculate the threshold. After setting 0 (black) to the pixel value above the threshold and setting 255 (white) to the pixel below above the threshold, binarization image was easily obtained. From Fig. 5b, we can see that there are too much signal edge noise points after the image was processed by the Sobel operator and Prewitt operator, with wide boundary signal region. Besides, there is no obvious boundary from both sides of the image after treated with the Roberts operator. Meanwhile, the image noise points were still not decreased after the process of the Log operator, while the one which treated with the improved operator can clearly see the left and right boundaries of the strip, with clear and narrow signal region boundary. Therefore, after comparison of these five algorithms (improved Sobel convolution operator algorithm, Sobel algorithm, Prewitt algorithm, Log algorithm, and Roberts algorithm), apparently, the improved Sobel convolution operator algorithm was the best one which derived a clear boundary and had less noise point.

The following step was to get the boundary coordinate of T and C lines through the binarization image. By calculating the left and right limits of test area, the coordinate in the *Y*-axis of the T/C line could be calibrated by two horizontal lines as shown in Fig. 5c. To confirm the accurate coordinate value, center expansion method (points of *ic* and *id* in Fig. 5c) and extreme method (points of *ia* and *ib* in Fig. 5c) were compared, which showed the former algorithm was much more effective. More noise signal existed beyond the area of the *ia* and *ib*. The procedure of the proposed algorithm is listed below.

1. The binarization image was the *M × N* matrix. Calculating the sum of every column pixel (5) in binarization image as the function *X(i)*, the function *X(i)* was displayed in Fig. 5c.
  5 $$ X(i)={\displaystyle {\sum}_{j=0}^M} S\left({x}_i,{y}_j\right) $$
  Obtaining the two extreme points (points of *i*a and *ib* in Fig. 5c) through Eqs. (6) and (7).
  6 $$ {X}_{\mathrm{extr}1}=\begin{array}{c}\hfill N/2\hfill \\ {}\hfill i=0\hfill \end{array}\mathrm{Max}\left( X(i)\right)= X\left(\mathrm{ia}\right) $$
  7 $$ {X}_{\mathrm{extr}2}=\kern1.44em \begin{array}{c}\hfill N\hfill \\ {}\hfill i= N/2\hfill \end{array}\mathrm{Max}\left( X(i)\right)= X\left(\mathrm{ib}\right) $$
  After then, the half of the smaller extreme value (points *X(ia)* and *X(ib)* in Fig. 5c) was set as the new threshold value through Eq. (8).
  8 $$ T=\frac{1}{2}\mathrm{M}\mathrm{i}\mathrm{n}\left({X}_{\mathrm{etr}1}+{X}_{\mathrm{extr}2}\right) $$
2. From the middle to both sides in horizontal axis, the value in the *X(i)* was compared with the threshold. Once the value was bigger than the threshold, the corresponding points (*ic*, *id*) were established through Eqs. (9) and (10).

9 $$ \begin{array}{l}\mathrm{from}\kern0.24em  k= N/ 2\;\mathrm{to}\ 0\\ {}\kern1.32em \mathrm{if}\; X(k)> T\kern0.36em \\ {}\kern2.4em  ic= k\\ {}\kern2.28em \mathrm{break};\\ {}\kern1.56em \mathrm{end}\\ {}\kern1.2em \mathrm{End}\end{array} $$

10 $$ \begin{array}{l}\mathrm{from}\; k= N/2\;\mathrm{to}\; N\\ {}\kern2.28em \mathrm{if}\; X(k)> T\\ {}\kern2.76em  id= k\\ {}\kern2.76em \mathrm{break};\\ {}\kern2.4em \mathrm{end}\\ {}\kern0.96em \mathrm{end}\end{array} $$

Through the extreme method, the two extreme points (*ia* and *ib*) of the coordinate position are acquired. Meanwhile, the center expansion method was used to calculate and to get much more accuracy value (*ic* and *id*) of (9) and (10).

As we know, T and C lines were two rectangular areas. By obtaining the left and right edge coordinates (points of *ic* and *id* in Fig. 5c) of the T/C line, the efficiency of the detection area was identified. The next work was to calculate the other two sides, as shown in Fig. 5d.

1. According to the varying degrees of definition in images, two methods were used. The first one is directly summing each row of the pixel values to obtain a one-dimensional array aim at obvious T/C lines.
  11 $$ Y(j)={\displaystyle {\sum}_{i=0}^N\; S\left({x}_i,{y}_j\right)} $$
2. Through calculating four extreme values (12–17), the extreme points (*c1*, *c2*, *t1*, *t2*) corresponded to the boundary coordinate of T and C lines.

12 $$ {Y}_{\mathrm{extr}}(k)=\left\{ Y(k)\left| Y(k)\right.{>}_{j= k-20}^{k-1} Y(j), Y(k){>}_{j= k+1}^{k+20} Y(j),20\le k\le N-20\right\} $$

13 $$ {Y}_{\mathrm{extr}}(k1)>{Y}_{\mathrm{extr}}(k2)>{Y}_{\mathrm{extr}}(k3)>{Y}_{\mathrm{extr}}(k4)> $$

14 $$ t 1= \min \left( k1, k2, k3, k4\right) $$

15 $$ t2=\left\{ \min \left( k1, k2, k3, k4\right), t2\ne t1\right\} $$

16 $$ C2= \max \left( k1, k2, k3, k4\right) $$

17 $$ C1=\left\{ \max \left( k1, k2, k3, k4\right), C1\ne C2\right\} $$

3. Aiming at unobvious T and C lines, the second method was to contrast the four extreme values (*c1*, *c2*, *t1*, and *t2*) and statistic of the averaging boundary coordinates of the T/C line in the previous experiments and then revised them.

Combining the two methods, the effective area of the test strip could be automatically identified, which was a great progress compared to other detection devices [32].

### Extraction for Detection Image Signals

**Fig. 6:**
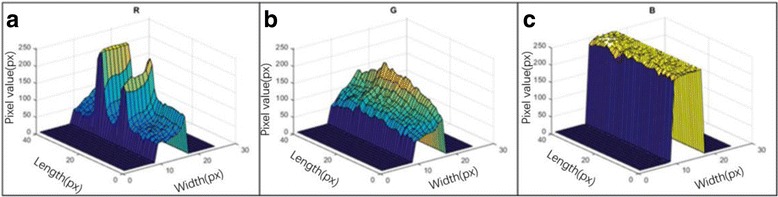
RGB channel pixel values of the fluorescent strip. **a** *Red* (R) channel. **b** Green (G) channel. **c** *Blue* (B) channel

According to different strips labeled by different NPs, we designed two methods to extract the signal of the strips: color strip and fluorescent strips. In the color strip, the pixel value of the red, green, and blue (RGB) three channels was extracted as the strip signal in T line and C line of the original image because every channel of the RGB three channels has indicated the intensity of the signal. In the fluorescent strips, only the red channel was extracted as the fluorescent intensity signal. From Fig. 6, we can see the green and blue channels could not indicate the fluorescent intensity and even had a negative effect on the signal extraction. So, the pixel value of the red channel was appropriate as the fluorescent intensity signal.

### Detection of Clinical Samples

The system was used to test the clinical samples. HCG and CEA samples (30 positive and 20 negative) were collected from a hospital. All the samples were pretreated with the treating fluid which was prepared by our group’s members. A double-blind test was used in this experiment. In the clinical samples, electrochemiluminescence assay was used to determined human HCG and commercial ECLIA kit (Roche Cobase 601) was the reference for CEA test. If one truly positive sample in the clinical samples was negatively detected by our method, the result was defined as false negative, and vice versa. The detection results were shown in Table 1. The sensitivity and specificity of the device were 97 and 100% for HCG and were 97 and 95% for CEA, respectively. From Table 1, it is demonstrated that the detecting system had a high sensitivity and specificity.

**Table 1:** Clinical test of HCG and CEA

Sample	Sample size	Positive	Negative	Validity
HCG	30(+)	29	1	Sensitivity 97%
	20(−)	0	20	Specificity 100%
CEA	30(+)	29	1	Sensitivity 97%
	20(−)	1	19	Specificity 95%

### Accuracy of the System

**Fig. 7:**
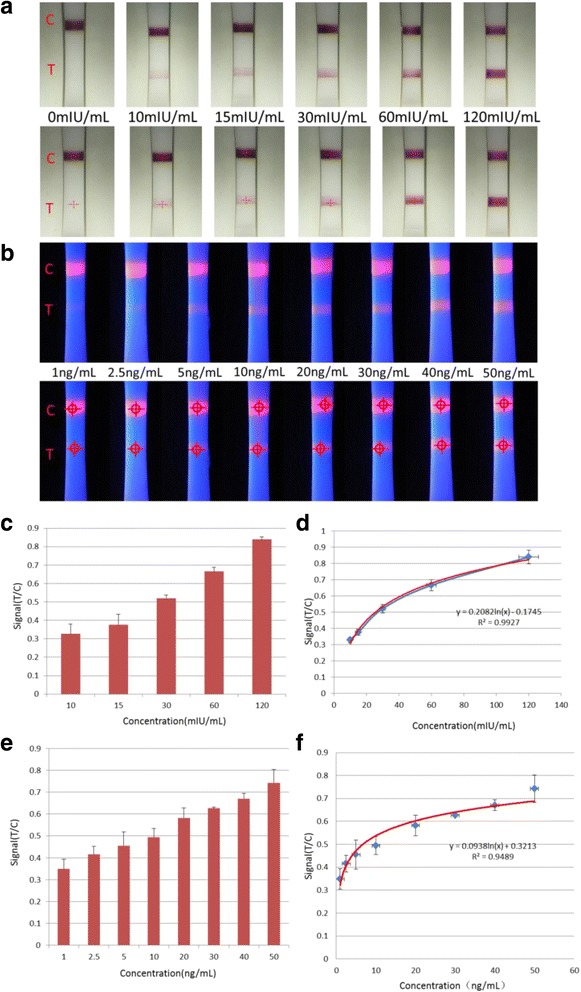
**a** Different concentrations of the colloidal gold strips for HCG. **b** Different concentration of the fluorescence strips for CEA. **c** Colloidal gold strip signal intensity. **d** Standard curve for quantitative detection of HCG. **e** Fluorescent strip signal intensity. **f** Standard curve for quantitative detection of CEA

In order to confirm the system accuracy, different bio-reagent concentration of the test strips was applied and the results were shown in Fig. 7. Figure 7a was the colloidal gold strips detection for HCG with six concentrations of analyte standards (0, 10, 15, 30, 60, and 120 mIU/mL) and Fig. 7b was the fluorescence strips detection for CEA with eight concentration of standard antigen (1, 2.5, 5, 10, 20, 30, 40, and 50 ng/mL). All the samples were prepared in a standard dilution buffer. Different concentration of the strips were detected for 20 times. From the results, we can see the areas of C and T lines were automatically marked correctly by the red circle even the vague shape of T line. Extracting the signal of the tagged area in the strips and calculating the ratio of T and C could further improve the sensitivity and the specificity of the system.

After recording the signal, the standard curve was obtained by plotting the linearity of T/C against the concentration of HCG or CEA, as represented by the equation in Fig. 7d, f. With an increase in concentration, more antigens were captured in the T line, leading to an increase in the T/C ratio. Meanwhile, the linear correlation coefficient (*R* ^2^) values were relatively high, and the ideal detection limit of the system was found to be 2.3 mIU/mL for HCG and 0.037 ng/mL for CEA.

### Repeatability and Stability of the System

**Fig. 8:**
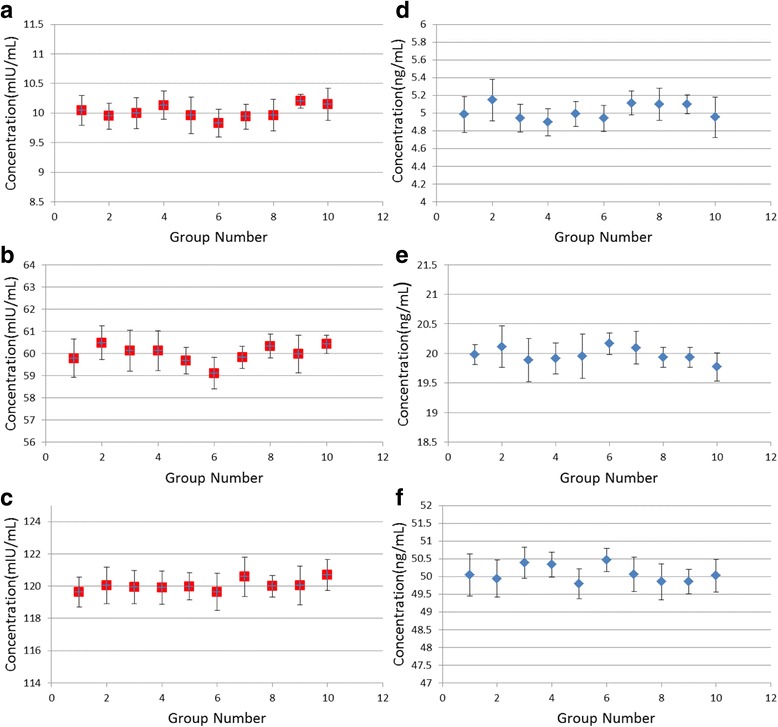
**a**–**c** The repeatability of colloidal strips for HCG. **a** The concentration of HCG was 10 mIU/mL. **b** The concentration of HCG was 60 mIU/mL. **c** The concentration of HCG was 120 mIU/mL. **d**–**f** The repeatability of fluorescent strip for CEA. **d** The concentration of CEA was 5 ng/mL. **e** The concentration of CEA was 20 ng/mL. **f** The concentration of CEA was 50 ng/mL

The different light intensities and strip locations caused slight variations in the acquired image. To test the repeatability of the system, the same concentration of ten strips was detected repeatedly and every strip was inserted into the system for 10 times within 5 min. In order to evaluate the stability of the system, immunochromatographic strips containing three different concentrations (10, 60, and 120 mIU/mL for HCG and 5, 20, and 50 ng/mL for CEA) were tested by the system. This test was performed to detect the error tolerance of the variation, and the results were presented in Fig. 8. The variance was calculated by the results after tested for 10 times at each strip. For HCG, 10, 60, 120 mIU/mL concentrations corresponding to the detection of the standard deviation were respectively 2.63, 1.37, 0.91%. For CEA, 5, 20, 50 ng/mL concentrations corresponding to the detection of the standard deviation were respectively 3.85, 1.45, and 1.05%. The variances of the whole test results were 1.6% (colored strip) and 2.1% (fluorescent strip), respectively. From the results, we can conclude that stability of the system is proportional to the sample concentration, and it presented the similar values from each measurement, thus proving the good repeatability of the system.

## Conclusions

In summary, a novel smartphone-based dual-modality imaging system was developed, which could quantitatively detect color or fluorescent lateral flow immunochromatographic strip (ICTS). Reasonable algorithms were applied to calculate the boundary localization and improve the accuracy of extracting signal, which achieved the high-signal intensity and sensitivity. The performance of the system was tested using different samples, which presented satisfactory results. Since there are many connected objects designed to link up with smartphone-controlled system and big data to realize bio-analysts and personalized healthcare monitoring and management, further work will focus on the development of a portable and versatile imaging system based on smartphone for detection in POCT in the near future.
